# A novel cold-active β-D-galactosidase from the *Paracoccus *sp. 32d - gene cloning, purification and characterization

**DOI:** 10.1186/1475-2859-10-108

**Published:** 2011-12-13

**Authors:** Anna Wierzbicka-Woś, Hubert Cieśliński, Marta Wanarska, Katarzyna Kozłowska-Tylingo, Piotr Hildebrandt, Józef Kur

**Affiliations:** 1 Department of Microbiology and Immunology, Faculty of Biology, University of Szczecin, Felczaka 3c, 71-412 Szczecin, Poland; 2 Department of Microbiology, Gdańsk University of Technology, Narutowicza 11/12, 80-233 Gdańsk, Poland; 3 Department of Pharmaceutical Technology and Biochemistry, Gdańsk University of Technology, Narutowicza 11/12, 80-233 Gdańsk, Poland

**Keywords:** Cold-active β-D-galactosidase, *Paracoccus *sp. strain 32d, lactose hydrolysis, cold-adapted microorganisms

## Abstract

**Background:**

β-D-Galactosidases (EC 3.2.1.23) catalyze the hydrolysis of terminal non-reducing β-D-galactose residues in β-D-galactosides. Cold-active β-D-galactosidases have recently become a focus of attention of researchers and dairy product manufactures owing to theirs ability to: (i) eliminate of lactose from refrigerated milk for people afflicted with lactose intolerance, (ii) convert lactose to glucose and galactose which increase the sweetness of milk and decreases its hydroscopicity, and (iii) eliminate lactose from dairy industry pollutants associated with environmental problems. Moreover, in contrast to commercially available mesophilic β-D-galactosidase from *Kluyveromyces lactis *the cold-active counterparts could make it possible both to reduce the risk of mesophiles contamination and save energy during the industrial process connected with lactose hydrolysis.

**Results:**

A genomic DNA library was constructed from soil bacterium *Paracoccus *sp. 32d. Through screening of the genomic DNA library on LB agar plates supplemented with X-Gal, a novel gene encoding a cold-active β-D-galactosidase was isolated. The *in silico *analysis of the enzyme amino acid sequence revealed that the β-D-galactosidase *Paracoccus *sp. 32d is a novel member of Glycoside Hydrolase Family 2. However, owing to the lack of a BGal_small_N domain, the domain characteristic for the LacZ enzymes of the GH2 family, it was decided to call the enzyme under study 'BgaL'. The *bgaL *gene was cloned and expressed in *Escherichia coli *using the pBAD Expression System. The purified recombinant BgaL consists of two identical subunits with a combined molecular weight of about 160 kDa. The BgaL was optimally active at 40°C and pH 7.5. Moreover, BgaL was able to hydrolyze both lactose and *o*-nitrophenyl-β-D-galactopyranoside at 10°C with *K*_m _values of 2.94 and 1.17 mM and *k*_cat _values 43.23 and 71.81 s^-1^, respectively. One U of the recombinant BgaL would thus be capable hydrolyzing about 97% of the lactose in 1 ml of milk in 24 h at 10°C.

**Conclusions:**

A novel *bgaL *gene was isolated from *Paracoccus *sp. 32d encoded a novel cold-active β-D-galactosidase. An *E. coli *expression system has enabled efficient production of soluble form of BgaL *Paracoccus *sp. 32d. The amino acid sequence analysis of the BgaL enzyme revealed notable differences in comparison to the result of the amino acid sequences analysis of well-characterized cold-active β-D-galactosidases belonging to Glycoside Hydrolase Family 2. Finally, the enzymatic properties of *Paracoccus *sp. 32d β-D-galactosidase shows its potential for being applied to development of a new industrial biocatalyst for efficient lactose hydrolysis in milk.

## Background

Cold-active enzymes found in cold-adapted organisms thriving in Earth's polar regions and other areas, where the mean annual temperature is below 5°C, offer a potential for the development of new industrial applications. Employing cold-active enzymes in the food industry reduces the risk of contamination by mesophilic microorganisms, allowing inactivation of them at moderate temperatures and changes in the taste and nutritional values of the foodstuffs being produced to be avoided [1].

Cold-active β-D-galactosidases primarily can be used in dairy industry for the production of lactose free milk for people afflicted with lactose intolerance. Besides eliminating nutritional problem, the low temperature of lactose hydrolysis in milk with an optimum temperature at approximately 10°C offer some other important advantages: (*i*) the lactose hydrolysis can run during shipping and storage of milk that shortening the entire production process (save energy), (*ii*) eliminating any mesophilic microflora contamination, and (*iii*) allow the formation of nonenzymatic browning products formed at higher temperatures to be avoided. On the other hand, there are also technological reasons for removing of lactose from milk. The lactose hydrolysis in milk decreases its hydroscopicity, as well as facilitating the suppression of lactose crystallization in sweet condensed milk and ice creams production processes. Furthermore, the enzymatic hydrolysis can be used to remove lactose from the whey generated in the cheese production process. The conversion of the lactose in whey to glucose and galactose, which are more fermentable sugars than lactose allow to reduces the water pollution related to the dairy industry [2].

In addition, cold-active β-D-galactosidases can be also used for the synthesis of oligosaccharides. Oligosaccharides are water soluble and mildly sweet in comparison with the commonly used mono- and disaccharides. Their relatively low sweetness is useful in food production where enhancement of other food flavors is desirable. Moreover, some oligosaccharides promote the proliferation of bifidobacteria in the colon, thus suppressing the growth of undesirable bacteria [3].

An ideal cold-active β-D-galactosidase for treating milk should work well at approximately 10°C; be highly active at pH 6.7-6.8; not be inhibited by Na^+ ^and Ca^2+ ^ions or galactose and glucose, and be specific for lactose. It is important to note that currently applied to lactose hydrolysis, the mesophilic *Kluyveromyces lactis *β-D-galactosidase (e.g. commercially available Lactozym - Novo Nordisk) has a temperature optimum of approximately 50°C and displays poor activity below 20°C. Therefore, in recent years, a great deal of effort has been invested in the isolation and characterization of novel cold-active β-D-galactosidases from different sources. These have mainly been bacterial enzymes isolated from *Arthrobacter *sp. B7 [4-6], *Arthrobacter *sp. C2-2 [3], *Arthrobacter *sp. SB [7], *Arthrobacter psychrolactophilus *strain F2 [1,8], *Arthrobacter *sp. 32c [9], *Arthrobacter *sp. 20B [2], *Flavobacterium *sp. 4214 [10]*Pseudoalteromonas *sp. 22b [11-13], *Pseudoalteromonas haloplanktis *[14], *Pseudoalteromonas *sp. TAE 79b [15], *Rahnella aquatilis *14-1 [16], *Planococcus *sp. SOS orange [17], *Planococcus *sp. L4 [18], and *Carnobacterium piscicola *strain BA [19]. In contrast the authors have found only a few reports of cold-active β-D-galactosidases isolated from other sources: yeast *Guehomyces pullulans *[20] and a soil metagenomic DNA library [21]. Hitherto, most of the cold-active β-D-galactosidases previously reported showed optimum temperature of activity at approximately 25-40°C, and only LacZ *Arthrobacter psychrolactophilus *strain F2 revealed optimum temperature of activity at 10°C. Generally, the most of above-mentioned cold-active β-D-galactosidases reveal high efficiency of the lactose hydrolysis in milk at low temperature, however, to the best of our knowledge, none of them is used as industrial biocatalyst so far.

Our previous studies on cold-active β-D-galactosidases isolated from *Pseudoalteromonas *sp. 22b [11-13] and *Arthrobacter *sp. 20B [2] revealed the factors that limit their usefulness as biocatalysts for industrial lactose hydrolysis in milk. These factors are, first, the insufficient efficiency of the production recombinant form of the cold-active LacZ *Pseudoalteromonas *sp. 22b β-D-galactosidase in *E. coli *expression system [12]. Secondly, the LacZ *Arthrobacter *sp. 20B [2] revealed the low stability of purified enzyme, that is the major disadvantage of using this cold-active β-D-galactosidase as biocatalyst, owing to the problems with providing the proper conditions for long storage of this enzyme.

The present study was conducted in order to carry out the molecular and enzymatic characterization of the cold-active recombinant β-D-galactosidase of *Paracoccus *sp. 32d. What is especially important, the active form of the recombinant enzyme was effectively produced by means of the *E. coli *expression system and the purified enzyme was stable and showed a high efficiency of lactose hydrolysis in milk. Moreover, to the best of the author's knowledge, this is the first report on characterization of β-D-galactosidase isolated from genus *Paracoccus*.

## Results

### Characterization and identification of the strain 32d

Strain 32d was Gram-negative, aerobic, non motile and rod-shaped. On LAS agar, this strain formed small, round, smooth, dark orange colonies with a diameter of 1-2 mm. The optimal growth temperature was 20°C and growth was very poor below 5°C and above 30°C. In contrast to β-D-galactosidase activity, lipase/esterase, amylase and protease activities were absent. Glucose, galactose and lactose were utilized.

**Figure 1:**
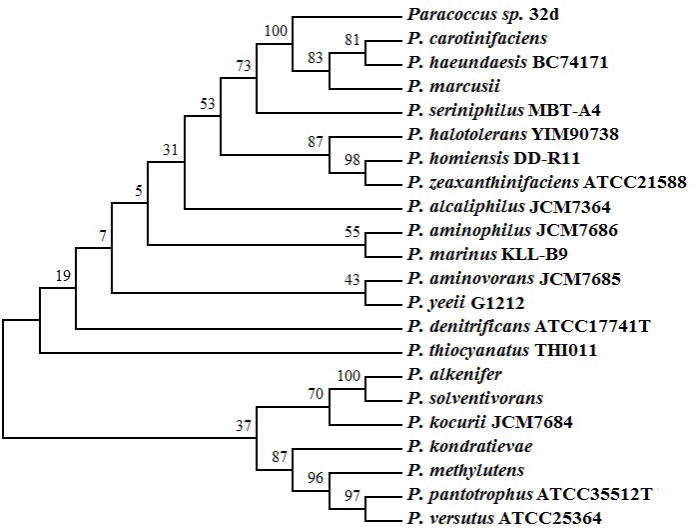
**Phylogenetic tree based on a neighbor-joining analysis of the 16S rDNA gene of strain 32d and closely related *Paracoccus *species **Gene sequences from the following organisms were used (the numbers in parentheses are the GenBank accession numbers): *P. alcaliphilus *JCM7364 (D32238), *P. alkenifer *(Y13827), *P. aminophilus *JCM7686 (D32239), *P. aminovorans *JCM7685 (D32240), *P. carotinifaciens *(AB006899), *P. denitrificans *ATCC17741T (Y16927), *P. haeundaesis *BC74171 (AY189743), *P. halotolerans *YIM90738 (DQ923133), *P. homiensis *DD-R11 (DQ342239), *P. kocurii *JCM7684 (D32241), *P. kondratievae *(AF250332), *P. marcusii *(Y12703), *P. marinus *KLL-B9 (AB185959), *P. methylutens *(AF250334), *P. pantotrophus *ATCC35512T (Y16933), *P. seriniphilus *MBT-A4 (AJ428275), *P. solventivorans *(Y13826), *P. thiocyanatus *THI011 (D32242), *P. versutus *ATCC25364 (Y16932), *P. yeeii *G1212 (AY014173), and *P. zeaxanthinifaciens *ATCC21588 (AF461158). Bootstrap 1000.

An alignment of the 16S rDNA gene sequence of the isolate 32d (GenBank, accession number GU111730.1) with the appropriate sequences available in the Ribosomal Database Project and the GenBank database, demonstrated that the isolate 32d should be classified as a *Paracoccus *sp. (Figure 1) and that its closest relative is *Paracoccus marcusii *(99% identity, 97% query coverage).

### Cloning the β-_D_-galactosidase gene from *Paracoccus *sp. 32d and analysis of its nucleotide sequence

The *Paracoccus *sp. 32d genomic DNA library was prepared in *E. coli *LMG194 and screened for the colonies that exhibited β-D-galactosidase activity. Finally, two positive transformants were selected as blue colonies on plates containing X-Gal (a chromogenic substrate for β-D-galactosidase). Both transformants were carrying the same recombinant plasmid with a *Bgl*II/*Sal*I-cleaved genomic DNA fragment of nearly 5.5 kb. Subsequently, one of these recombinant plasmids (pBAD/insβ1) was selected for further study. Sequence data from the pBAD/insβ1 insert revealed an open reading frame of 2,193 bp encoding protein, which shares an average homology (63%) with a β-D-galactosidase from *Sinorhizobium fredii *NGR234 (NCBI Accession No. ACP21732). The β-D-galactosidase encoded the ORF under analysis contained 731 amino acids residues, giving a calculated molecular weight of 81,750.4 Da and a theoretical pI of 5.28 (ProtParam; ExPASy Proteomics Server).

### The primary structure of *Paracoccus *sp. 32d β-_D_-galactosidase

**Figure 2:**
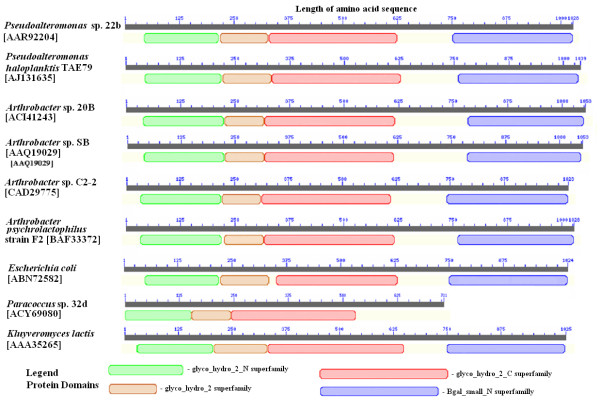
**Topographic presentation of Pfam domains for selected LacZ β-D-galactosidases and *Paracoccus *sp. 32d β-D-galactosidase BgaL **The domains presented were suggested by the Pfam database http://www.sanger.ac.uk/software/Pfam and are indicated by different colors. The numbers in parentheses are the GenBank accession numbers.

**Figure 3:**
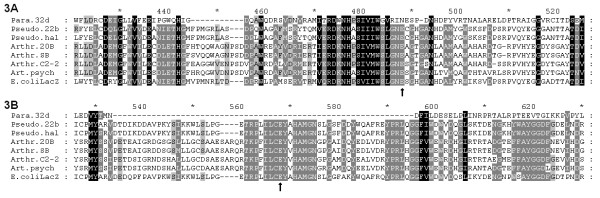
**Alignment of the amino acids sequences of β-D-galactosidase (A) acid-base active sites, and (B) the consensus nucleophilic region of the selected LacZ enzymes and *Paracoccus *sp. 32d β-D-galactosidase **The numbers in parentheses are GenBank accession numbers. Proposed active site glutamic acid residues are indicated by the black arrows. Para.32d - *Paracoccus *sp. 32d BgaL (ACY69080), Pseudo.22b - *Pseudoalteromonas *sp. 22b LacZ (AAR92204), Pseudo.hal - *Pseudoalteromonas haloplanktis *TAE79 LacZ (AJI31635), Arthr. 20B - *Arthrobacter *sp. 20B LacZ (ACI41243), Athr. SB - *Arthrobacter *sp. SB LacZ (AAQ19029), Arthr.C2-2 - *Arthrobacter *sp. C2-2 LacZ (CAD29775), Art.psych - *Arthrobacter psychrolactophilus *strain F2 LacZ (ABN72582) and E.coliLacZ - *E. coli *LacZ (ABN72582).

A computer analysis of the amino acid sequence deduced for *Paracoccus *sp. 32d β-D-galactosidase, conducted using the InterProScan program http://www.ebi.ac.uk/Tools/InterProScan/ showed that it consisted of a carbohydrate-binding domain (1-151 aa residues), a immunoglobulin-like β-sandwich/β-D-galactosidase/glucuronidase domain (153-243 aa residues), and a single catalytic domain (245-528 aa residues). Moreover, this comparison revealed the lack of Bgal_small_N domain at the C-terminus of *Paracoccus *sp. 32d β-D-galactosidase, a domain characteristic of LacZ enzymes (Figure 2). On the basis of sequence comparisons carried out by means of homology and hydrophobic cluster analysis [22], the enzyme from *Paracoccus *sp. 32d was classified into the Glycoside Hydrolase Family 2 which comprises the well-characterized LacZ β-D-galactosidases, such as *E. coli *LacZ β-D-galactosidase. However, the comparison of *Paracoccus *sp. 32d β-D-galactosidase sequence with cold-active LacZ β-D-galactosidases and *E. coli *LacZ enzyme sequences revealed a slight sequence homology in the vicinity of the catalytic glutamic acid residue present in the putative Acid/Base sites of LacZ enzymes (Figure 3A). Moreover, the comparison failed to find any homology with the consensus nucleophilic region of the LacZ enzymes (Figure 3B).

### Expression and purification of *Paracoccus *sp. 32d β-_D_-galactosidase

**Figure 4:**
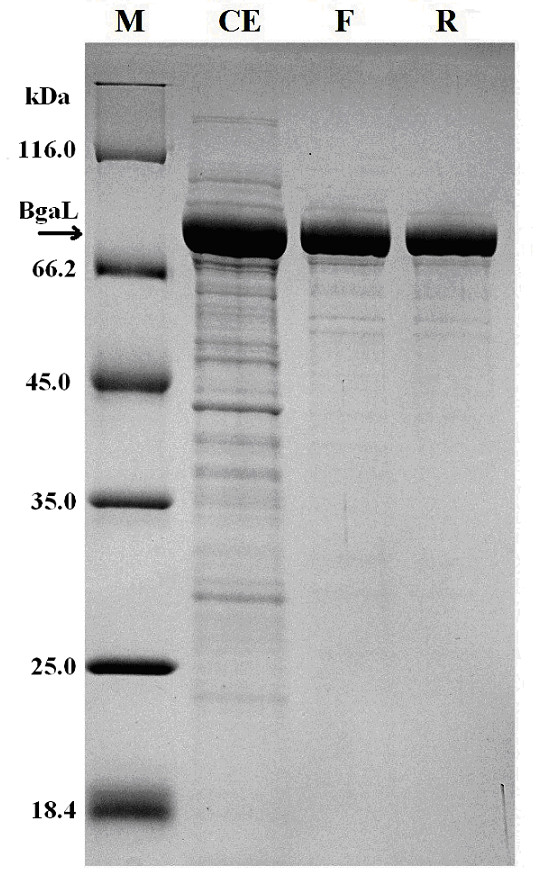
**SDS-PAGE (12% polyacrylamide gel) protein profiles of fractions collected after successive purification steps carried out on recombinant *Paracoccus *sp. 32d β-D-galactosidase from *E. coli *strain LMG194 **Lane M - protein molecular weight marker; lane CE - cell extract; lane F - pooled fraction after Fractogel EMD DEAE chromatography; lane R - pooled fraction after Resource Q chromatography.

The arabinose-inducible promoter of the pBAD-Myc-His A plasmid was used for the expression of the *Paracoccus *sp. 32d β-D-galactosidase gene in *E. coli *LMG194 cells. The highest enzyme production yields were achieved by adding L-arabinose to a final concentration of 0.2% w/v, at A_600 _0.5-0.55 and by further cultivation for 8 h at 30°C. The enzyme was purified by using the two-step procedure, presented in Table 1. Following this procedure, the enzyme was ~96% pure (densitometric analysis; software ImageJ v 1.44I) as determined by SDS-PAGE (Figure 4) and had an estimated apparent molecular mass of 80 kDa corresponding to the expected molecular mass calculated from the BgaL amino acid sequence. The relative molecular mass of recombinant BgaL, which was determined by gel filtration was 161 kDa suggesting that the *Paracoccus *sp. 32d β-D-galactosidase is a dimer protein.

**Table 1:** Purification of recombinant *Paracoccus *sp. 32d β-D-galactosidase

Purification step	Total protein (mg)	Total activity (U)	Specific activity (U/mg)	Purification fold	Yield (%)
Cell extract	266.1	6213	23.35	1.0	100
Fractogel EMD DEAE	152.6	6053	39.67	1.7	97
ResourceQ	105.6	4328	40.98	1.8	72

Finally, the purified enzyme was divided into two aliquots. One of the aliquots was dialyzed against a Tris-HCl buffer (20 mM, pH 7.3) and then used for protein crystallization experiments (data not shown).

What it is important to note is that, the activity of the purified β-D-galactosidase was depended on the buffer used to purify or store the enzyme. We compared the enzymatic activity of BgaL against *o*-nitrophenyl-β-D-galactopyranoside as a substrate in a sodium phosphate buffer (20 mM, pH 7.3) and the Tris-HCl buffer (20 mM, pH 7.3), respectively. The *Paracoccus *sp. 32d β-D-galactosidase revealed significantly higher activity in the sodium phosphate buffer than in the Tris-HCl buffer. The relative activity of β-D-galactosidase in the Tris-HCl buffer was only 57% of the maximum enzymatic activity of BgaL in sodium phosphate buffer, respectively. Thus, we decided to characterize the enzymatic properties of BgaL with using the sodium phosphate buffer.

On the other hand the results presented in Table 1 reveal that the second purification step had a low efficiency. Moreover, the densitometric analysis of SDS-PAGE gel stained with Coomassie Brilliant blue (Figure 4) revealed that the BgaL enzyme was ~93% pure after the first purification step. Therefore, the one step purification procedure of BgaL is the rationale way to reduce the cost of BgaL purification on large scale, important for the production of the enzyme for industrial applications.

### Properties of *Paracoccus *sp. 32d β-_D_-galactosidase

A study of the substrate specificity of purified *Paracoccus *sp. 32d enzyme was performed by comparing its enzymatic activity against the *o*-nitrophenyl-β-D-galactopyranoside (ONPG) and a variety of *p*-nitrophenyl (PNP)-β-glycoside substrates, respectively (Table 2). The *Paracoccus *sp. 32d enzyme revealed enzymatic activities specific to the β-D-galactosidase. The highest activity was found with lactose analogs ONPG. The activity with *p*-nitrophenyl-β-D-galactopyranoside and *p*-nitrophenyl-β-D-fucopyranoside as substrates were only 62% and 39% of that found with ONPG, respectively.

**Table 2:** Relative activity of purified *Paracoccus *sp. 32d β-D-galactosidase with various nitrophenyl-derived chromogenic substrates

Substrate	Relative activity (%)
*o*-nitrophenyl-β-D-galactopyranoside	100
*p*-nitrophenyl-β-D-galactopyranoside	62
*p*-nitrophenyl-β-D-fucopyranoside	39
*p*-nitrophenyl-β-D-galacturonide	1
*p*-nitrophenyl-β-D-glucopyranoside	< 0.01
*p*-nitrophenyl-β-L-arabinopyranoside	< 0.01
*p*-nitrophenyl-β-D-cellobioside	< 0.01
*p*-nitrophenyl-β-D-xylopyranoside	< 0.01
*p*-nitrophenyl-β-D-mannopyranoside	< 0.01
*p*-nitrophenyl-β-D-glucuronide	< 0.01
*p*-nitrophenyl-α-D-galactopyranoside	< 0.01

**Figure 5:**
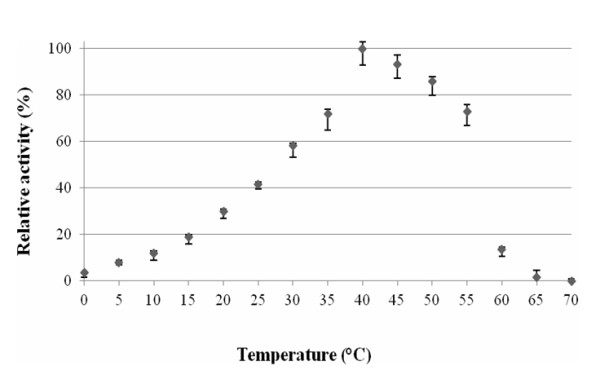
**The effect of temperature on the recombinant *Paracoccus *sp. 32d β-D-galactosidase activity**.

**Figure 6:**
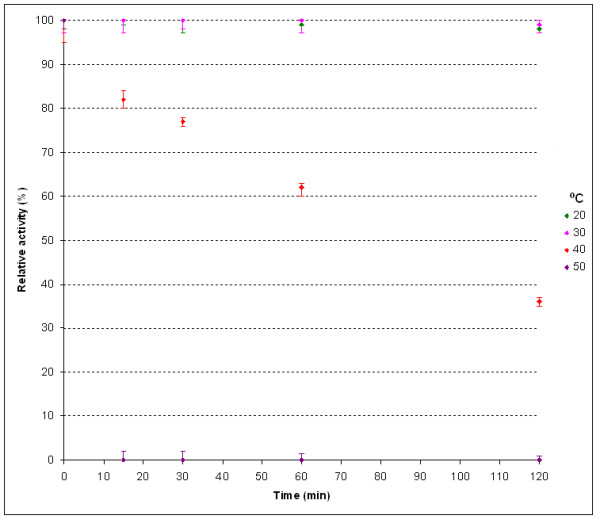
**The effect of temperature on the recombinant *Paracoccus *sp. 32d β-D-galactosidase stability**.

The thermodependency of the *Paracoccus *sp. 32d β-D-galactosidase activity was determined by assaying the enzyme activity at various temperatures from 0 to 70°C using ONPG as a substrate. The maximum activity enzyme shows at a temperature of 40°C (Figure 5). After 2 h incubation, the enzyme was thermostable at 30°C and below. However, the incubation at 50°C caused a rapid decrease of activity after 15 min incubation (Figure 6).

**Figure 7:**
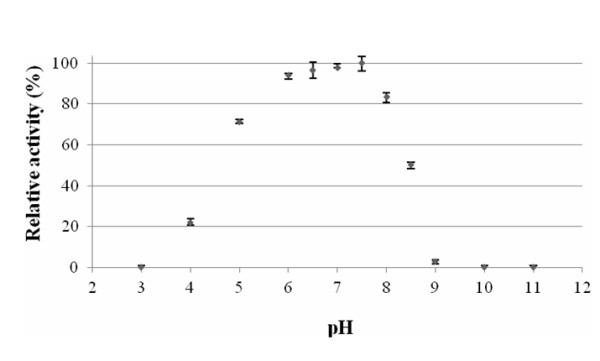
**The effect of pH on the recombinant *Paracoccus *sp. 32d β-D-galactosidase activity**.

**Figure 8:**
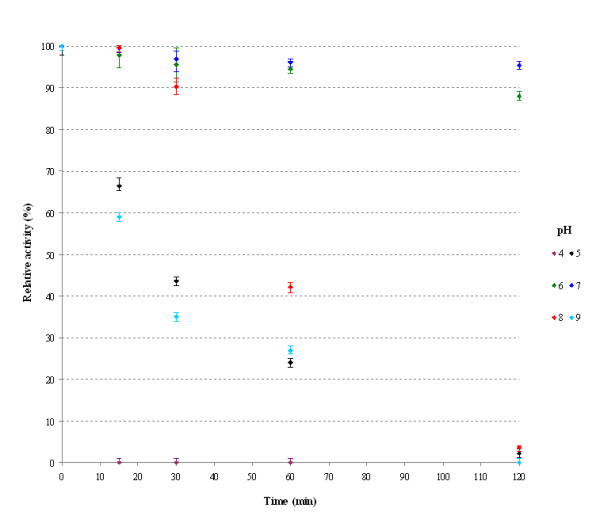
**The effect of pH on the recombinant *Paracoccus *sp. 32d β-D-galactosidase stability**.

The *Paracoccus *sp. 32d β-D-galactosidase revealed maximum activity at pH 7.5 and demonstrated above 80% of the maximum activity at a range of pH 6.0-8.0 (Figure 7). The enzyme was stable at pH 6.0 and 7.0 (~90% of maximum activity) after 2 h incubation, but it rapidly lost activity at pH 4.0 after 15 min incubation (Figure 8).

**Figure 9:**
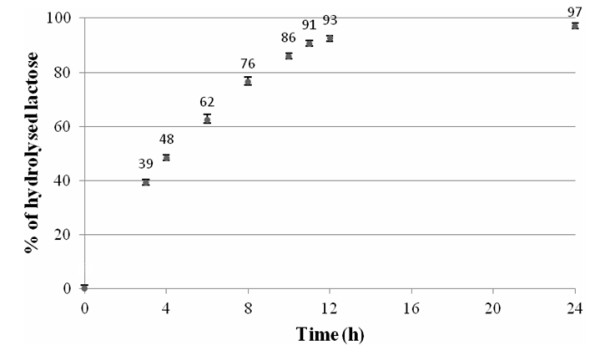
**Hydrolysis of milk lactose by 1U of *Paracoccus *sp. 32d β-D-galactosidase as a function of time-course**.

As shown in Table 3 the activity of the enzyme against ONPG as a substrate was slightly enhanced by K^+ ^ions and 2-mercaptoetanol and was unaffected by EDTA. It was slightly inhibited by Mg^2+ ^ions, dithiothreitol and urea, respectively and was also partially inhibited by Ca^2+^, Mn^2+^, Ni^2+^, Co^2+ ^ions and strongly inhibited by oxidized form of glutathione (GSSG). The strong inhibition effect of GSSG and the positive effect of 2-mercaptoetanol on the BgaL activity suggest the importance of Cys residues in this protein sequence. The Cys residues are uninvolved in the general mechanism of catalysis by β-D-galactosidases of the LacZ family [23]. However, the S-thiolation or oxidation of sulfhydryl group of some cysteine residues can lead to the conformational changes of BgaL that decrease its activity. The enzyme activity was also inhibited by glucose and galactose, the products of lactose hydrolysis. As shown in Table 4 the enzyme activity inhibition increases with increasing sugars concentrations and the glucose is a stronger inhibitor than galactose. On the other hand, 1 U of the enzyme was able to hydrolyze about 97% and 91% of the lactose in 1 ml of milk at 10°C in 24 h and 11 h, respectively (Figure 9).

**Table 3:** The effect of metal ions and selected reagents on *Paracoccus *sp. 32d β-D-galactosidase activity

Ions/Reagents (10 mM)	Residual activity (%)
None	100
K^+^	106
Mg^2+^	97
Ca^2+^	70
Mn^2+^	75
Ni^2+^	61
Co^2+^	81
EDTA	100
DTT	93
Glutathione oxidised	30
2-mercaptoethanol	107
Urea	98

**Table 4:** The effect of glucose and galactose on the recombinant *Paracoccus *sp. 32d β-D-galactosidase activity

Sugar concentration (mM)	Relative activity (%)	
	Glucose	Galactose
0	100 ± 1	100 ± 2
20	75 ± 1	83 ± 2
50	56 ± 1	67 ± 1
100	38 ± 2	52 ± 1
150	29 ± 1	39 ± 2

A lyophilized enzyme was stable for at least a year when it was stored desiccated at -20°C. The reconstituted enzyme (20 mM sodium phosphate buffer, pH 7.3) was effective in hydrolysis of ONPG (95 ± 2% of the starting enzymatic activity) or lactose (93 ± 2% of the starting enzymatic activity) at 20°C.

A freshly purified enzyme was used to determine the *K*_m_, *k*_cat _and *k*_cat_*/K*_m _values at 10, 20 and 30°C with ONPG and lactose as substrates, respectively. As shown in Table 5, the apparent *K*_m _values for lactose increase at temperatures higher and lower than 20°C. As a result, the enzyme's efficiency (*k*_cat_/*K*_m _ratio) for lactose is markedly affected at these temperatures, whereas this ratio is about two or four times higher at 20°C. On the other hand, the apparent *K*_m _values for ONPG are comparable at 10, 20 and 30°C, and adequate *k*_cat_/*K*_m _ratios increase constantly with an increasing temperature.

**Table 5:** Kinetic parameters of *Paracoccus *sp. 32d β-D-galactosidase

Substrate	Temperature (°C)	*K*_m _(mM)	*k*_cat _(s^-1^)	*k*_cat_/*K*_m _(s^-1^mM^-1^)
ONPG	10	1.17 ± 0.11	71.81 ± 2.11	61.38
	20	1.18 ± 0.06	138.3 ± 2.19	117.20
	30	0.99 ± 0.13	206.00 ± 6.92	208.08

Lactose	10	2.94 ± 0.39	43.23 ± 1.13	15.06
	20	1.16 ± 0.98	66.67 ± 5.6	57.47
	30	4.28 ± 0.57	140.00 ± 4.64	32.71

## Discussion

A novel β-D-galactosidase gene from *Paracoccus *sp. 32d was cloned from a genomic DNA library by means of functional screening on β-D-galactosidase indicator plates. A *Bgl*II/*Sal*I genomic DNA fragment bearing the β-D-galactosidase gene was sequenced, and an ORF encoding the β-D-galactosidase was found. Computer analysis of the BgaL amino acid sequence established that the *Paracoccus *sp. 32d enzyme belongs to Glycoside Hydrolase Family 2 (GH2). However, in contrast to other cold adapted β-D-galactosidases belonging to the GH2 family, the *Paracoccus *sp. 32d β-D-galactosidase possesses an acid-base active site slight similar to that typical of the LacZ family of β-D-galactosidase. However, it does not share the conserved nucleophilic site involved in catalysis that is typically found in this family of enzymes. Similar results for the same analysis were described by Gutshall et al. [5] for cold-active β-D-galactosidase, designated isozyme 12, and isolated from psychrotrophic *Arthrobacter *strain B7. However, what is important to note is that the sequence analysis of isozyme 12 revealed that this enzyme belongs to Glycoside Hydrolase Family 42.

Moreover, to the best of the authors' knowledge, the *Paracoccus *sp. 32d enzyme is the first dimeric cold-active β-D-galactosidase determined as belonging to the GH2 family. Hitherto, the cold-active β-D-galactosidases characterized and belonging to that family are tetrameric enzymes [2,3,7,11,14,15]. What is interesting to note is that, in size the *Paracoccus *sp. 32d β-D-galactosidase subunit (~80 kDa) is clearly smaller than the subunit sizes typical of the LacZ family of cold-active β-D-galactosidase, such as, for example, LacZ *Pseudoalteromonas *sp. 22b (~115 kDa) [11] or LacZ *Arthrobacter *sp. 20B (~116 kDa) [2]. The difference in size is caused by the lack of a BGal_small_N domain at the C-terminus of *Paracoccus *sp. 32d β-D-galactosidase (Figure 2). The BGal_small_N domain (pfam02929) is commonly found in β-D-galactosidases (Conserved Domains Database, NCBI). The catalytic and other domains typically found in LacZ enzymes are present in BgaL enzyme (Figure 2). The lack of the BGal_small_N domain make the *Paracoccus *sp. 32d β-D-galactosidase one of the smallest cold-active β-D-galactosidases (161 kDa, homodimer) to have been described to date. In comparison, the relative low molecular mass of native cold-active β-D-galactosidases have been reported for isozyme 14 of *Arthrobacter *sp. B7 (110 kDa, homodimer) [6], *Planococcus *sp. SOS Orange (150 kDa, homodimer) [17] and *Arthrobacter *sp. 32c (195 kDa, homotrimer) [9]. On the other hand, Hildebrand et al. [9] suggest that the low molecular mass of native β-D-galactosidase is crucial to the effective extracellular production of heterologous protein in yeast *Pichia pastoris*. Comparison of the authors' previous results for the production of LacZ (~490 kDa, homotetramer) *Pseudoalteromonas *sp. 22b in *E. coli *[12], with the analogous production efficiency of BgaL revealed that the low molecular mass of *Paracoccus *sp. 32d β-D-galactosidase also seems to be crucial for the effective production of this protein in *E. coli *cells.

ONPG is the preferred chromogenic substrate for BgaL (Table 2). Of interest is the fact that the many of the well-characterized cold-active β-D-galactosidases, isolated from *Pseudoalteromonas *sp. 22b [11], *Arthrobacter *sp. 20B [2], *Arthrobacter *sp. 32c [9], *Carnobacterium piscicola *BA (BgaB)* [19] and *Arthrobacter *sp. B7 isozyme 12* [5] and isozyme 14* [6] preferred *p*-nitrophenyl-β-D-galactopyranoside (PNPG) as a substrate (the asterisk means that the selected studies presented data only for *p*-Nitrophenyl (NP)-linked substrates).

*Paracoccus *sp. 32d β-D-galactosidase has an optimum temperature of approximately 40°C, and a low thermostability at over 30°C, and is active at a pH range of 6.0-8.0 with an optimum activity at 7.5. The comparable enzymatic properties were encountered in the other well-characterized cold-active β-D-galactosidases such as LacZ *Arthrobacter *sp. C2-2 [3], *Arthrobacter *sp. B7 isozyme 15 [4], and LacZ *Pseudoalteromonas *sp. 22b [11]. However, the enzymatic activity of the *Paracoccus *sp. 32d β-D-galactosidase at a temperature of 10°C is no more than around 15% of its maximum activity at optimum temperature (Figure 5). This is the one of the lowest relative activities for β-D-galactosidase at 10°C, as compared with the analogous enzymatic activities reported at this temperature for cold-active β-D-galactosidases such as LacZ *Arthrobacter *sp. C2-2 (~20%) [3], LacZ *Pseudoalteromonas *sp. TAE 79b (~40%) [15], BgaB *Carnobacterium piscicola *BA (~25%) [19], LacZ *Arthrobacter *sp. 20B (~70%) [2], LacZ *Arthrobacter *sp. SB (~70%) [7], LacZ *Arthrobacter *sp. C2-2 (~25%) [3], LacZ *Arthrobacter psychrolactophilus *strain F2 (~100%) [8] and LacZ *Pseudoalteromonas *sp. 22b (~24%) [11]. To the best of the authors' knowledge, the lowest enzymatic activity (~10% of its maximum activity at optimum temperature) for cold-active β-D-galactosidase at 10°C has been reported for the enzyme isolated from *Flavobacterium *sp. 4214 [10].

The study of the kinetic properties of BgaL against an ONPG and a lactose as substrates, revealed the different trends of changes in the relevant *k*_cat_*/K*_m _and *K*_m _values against these substrates at 10, 20 and 30°C, respectively (Table 5). The markedly higher affinity of the lactose to BgaL at 20°C (*K*_m _value) than at 10°C or 30°C indicate the molecular adaptation of the enzyme to effective catalysis (*k*_cat_*/K*_m _value) of this native substrate at the optimal temperature of growth for *Paracoccus *sp. 32d. In contrast, an enzymatic efficiency of BgaL against ONPG (synthetic analogous of lactose) was increasing constantly with an increasing temperature.

Generally, the enzymatic properties for BgaL seem to be somewhat removed from those of cold-active β-D-galactosidase which are ideal for the removal of lactose from milk where activity at refrigerated temperatures is critical. One of the most notable features of the BgaL enzyme is the inhibition of its hydrolytic activity by galactose and glucose, and Ca^2+ ^ions. What surprised the authors, however, was the high efficiency finding as regards the removal of lactose from milk by BgaL from *Paracoccus *sp. 32d (Figure 9), that was comparable with the analogous ones of cold-active β-D-galactosidases previously characterized by us and our former co-workers from IBT at the Technical University of Lodz [2,9,11,12,24]. It seems to be possible that the complex physicochemical properties of milk could have a positive effect on the enzymatic activity of *Paracoccus *sp. 32d β-D-galactosidase. For example, this study found the significant differences between the enzymatic activity of BgaL in the Tris-HCl buffer and the sodium phosphate buffer, respectively. The enzymatic activity of BgaL against ONPG as a substrate at the same pH and temperature was 47% higher in the sodium phosphate buffer than in the Tris-HCl buffer, respectively.

## Conclusions

This study presents the purification and characterization of a new β-D-galactosidase from *Paracoccus *sp. 32d. From the sequence analyses it is obvious that the BgaL enzyme is a member of the Glycoside Hydrolase Family 2. However, the sequence analysis of the BgaL enzyme reveal the lack of the BGal_small_N domain previously found in other cold-active β-D-galactosidases belong to GH2 family. Both the relatively low molecular weight of the *Paracoccus *sp. 32d BgaL enzyme, the efficient production of its recombinant soluble and active form in *E. coli *cells, and the efficient hydrolysis of lactose in milk suggested that *Paracoccus *sp. 32d β-D-galactosidase exhibits potential for the development of a new industrial cold-active biocatalyst.

## Methods

### Bacterial strains and cultivation conditions

The *Paracoccus *strain 32d from the Department of Microbiology GUT (Gdansk, Poland) collection of Antarctic microorganisms was isolated from the soil sampled in the neighborhood of the Henryk Arctowski Polish Antarctic Station at King George Island (Southern Shetlands, 62°10'S, 58°28'W). Strain 32d was cultivated in a modified Luria-Bertani medium: LBS (10 g peptone K, 5 g yeast extract, and 10 g sea salt per 1 L, pH 7.5) at 20°C.

*E. coli *LMG 194 (F_Δ*lacX74 galE galK thi rpsL *Δ*phoA *(PvuII) Δ*ara714 leu::Tn10*) cells were used for cloning and expression of the recombinant *Paracoccus *sp. 32d β-D-galactosidase. What was important to note is that due to the deletion of *lac *operon (Δ*lacX74*) the *E. coli *LMG 194 is *lac*Z deficient strain. *E. coli *strain was grown on LB medium (10 g peptone K, 5 g yeast extract, and 10 g NaCl per 1 L, pH 7.5) or on LB medium solidified with bacteriological agar at 37°C (for cloning and expression experiments) and 30°C (for expression experiments).

### Characterization and identification of the strain 32d

Growth properties were determined in LAS broth (10 g peptone K, 5 g yeast extract, 15 g bacteriological agar and 10 g sea salt per 1 L, pH 7.5). The strain 32d was tested by using minimal media containing 0.5% (w/v) of the glucose, galactose and lactose as a sole carbon source. The proteolytic, lipolytic and amylolytic activities of the analyzed strain were examined at 20°C, on plates holding nutrient agar, enriched with skimmed milk, tributyrin, and starch, respectively.

The genus of the strain 32d was assessed on the basis of the 16S rDNA gene sequence, amplified by PCR technique with primers fD1 and rP2 [11]. The 16S rDNA PCR product was sequenced using ABI 3730 xl/ABI 3700 sequencing technology (Genomed, Poland). The 16S rRNA gene sequence was compared with those from the Ribosomal Database Project and the NCBI database aligned using the MEGA 5.0 http://www.megasoftware.net/.

### General DNA manipulations

Restriction enzymes were purchased from Fermentas (Lithuania). The T4 DNA ligase was purchased from Epicentre (USA). Restriction enzymes and other DNA-modifying enzymes were used in accordance with the manufacturer's recommendations. The reagents for PCR were purchased from DNA-Gdańsk II (Poland). The kits for genomic DNA isolation (Genomic Mini) and plasmid DNA isolation (Plasmid Mini) were purchased from A&A Biotechnology (Poland).

### Genomic DNA library construction and β-D-galactosidase gene identification

The chromosomal DNA from *Paracoccus *sp. 32d cells was extracted using a Genomic Mini kit according to the protocol for gram-negative bacteria. The genomic DNA was digested using the *Bgl*II and *Sal*I endonucleases, and the resultant DNA fragments were purified by isopropanol precipitation protocol http://www.uccs.edu/~rmelamed/Lab/General%20Procedures/EthanolPrecip.html. The purified DNA fragments were ligated with T4 DNA ligase into the corresponding sites of pBAD/*Myc*-His A (Invitrogen, USA). The ligated DNA was transformed into *E. coli *LMG 194 and clones were selected on Luria-Bertani agar plates supplemented with ampicillin (0.1 mg ml^-1^), X-Gal (0.02 mg ml^-1^), and IPTG (0.1 mg ml^-1^). The ampicillin, IPTG and X-Gal were purchased from Sigma (USA). The plates were incubated at 30°C for 18 h and then transferred to 20°C. After 4 h of incubation at 20°C, the two recombinant colonies, producing β-D-galactosidase turned blue. Plasmid DNA from the positive transformants (blue colonies) was prepared using the Plasmid Mini kit (A&A Biotechnology, Poland). Samples with plasmid DNA were digested with selected restriction enzymes (Fermentas, Lithuania) to create restriction maps of the constructs under examination. DNA inserts sequencing was performed using ABI 3730 xl/ABI 3700 sequencing technology (Genomed, Poland). Sequence similarity analyses were carried out using the Basic Local Alignment Search Tool program and on the server at National Centre of Biotechnology, USA http://www.ncbi.nih.gov/blast. Nucleotide and deduced amino acids sequence analyses were performed with VNTI advanced 10 (Invitrogen, USA). The ORFs search was performed with the ORF Finder program http://www.ncbi.nlm.nih.gov/gorf/gorf.html. The ORF corresponding to the β-D-galactosidase was named the *bgaL *gene. BgaL protein sequence analysis and classification was conducted by means of the InterProScan software http://www.ebi.ac.uk/interpro/.

### Expression and purification of recombinant BgaL of *Paracoccus *sp. 32d

The expression vector pBAD/Myc-His A (Invitrogen, USA) was used for the expression of the *bgaL *gene of *Paracoccus *strain 32d in *E. coli *strain LMG 194. The *bgaL *gene was amplified using Forgal32d primer 5'-AAATC**ATGA****GGGTGACCCAGAAACTGAACCATGGC**-3' (containing the *Bsp*HI recognition site), and Revgal32d primer 5'-AAATGTCGAC**CTAGCCGACGGTGACCGTGGCC**-3' (containing the *Sal*I recognition site). The parts of the primer sequences given in boldface are complementary to the nucleotide sequences of the *Paracoccus *sp. 32d *bgaL *gene, while the recognition sites for the restriction endonucleases are underlined and were designed to facilitate cloning. The PCR fragment obtained was cloned into the *Nco*I and *Sal*I sites of pBAD/Myc-His A under the P_BAD _promoter, yielding pBAD/LacZ32d. The resultant recombinant plasmid was transformed into a competent cells of *E. coli *strain LMG 194. The transformants were grown in an LB medium (1 L) containing ampicillin (0.1 mg ml^-1^), and shaken, at 37°C and 200 rpm, to an optical density of 0.5-0.55 measured at 600 nm. The culture was then supplemented with L-arabinose (0.2% w/v) to induce the expression of the *bgaL *gene and grown for 8 h at 30°C. Next, the recombinant *E. coli *cells were harvested by centrifugation at 4600 × *g *for 15 min. The cell pellet was resuspended in 30 ml of the A buffer (0.02 M sodium phosphate buffer, pH 6.3, 0.1 M NaCl), and then the cells were then disrupted by sonication, and chilled on ice. The cell debris was collected by centrifugation at 13,000 × *g *for 20 min at 4°C and then the cell-free extract was applied onto Fractogel EMD DEAE column (Merck, Germany) previously equilibrated with the A buffer. An elution was carried out with a linear NaCl gradient (0.05-0.6 M) in the A buffer, and with a flow rate of 1 ml min^-1^. Afterwards, the purified β-D-galactosidase-active fractions were combined and dialyzed to the A buffer and applied onto a Resource Q column (Merck, Germany) previously equilibrated with the A buffer. An elution was carried out with a linear NaCl gradient (0.1-0.6 M) in the A buffer, and with a flow rate of 0.5 ml min^-1^. Finally, the β-D-galactosidase-active fractions, eluted within the a range of 0.3-0.35 M NaCl, were combined and dialyzed to the C buffer (0.02 M sodium phosphate buffer, pH 7.3).

### Estimation of molecular weight

The purified enzyme was applied onto a Superdex 200 10/300 GL gel-filtration column (Amersham Bioscience) pre-equilibrated with 50 mM sodium phosphate buffer, 150 mM NaCl (pH 7.0). Gel filtration was performed by means of high-performance liquid chromatography, with the same buffer as the eluent, and at a flow rate of 0.5 ml min^-1^, and the elution patterns were compared with those of the standard proteins. The standard proteins used were thyroglobulin (*M*_r _= 669,000 Da), apoferritin (*M*_r _= 440,000 Da), β-amylase (*M*_r _= 200,000 Da), alcohol dehydrogenase (*M*_r _= 150,000 Da), bovine serum albumin (*M*_r _= 66,000 Da), and carbonic anhydrase (*M*_r _= 29,000 Da).

### Protein determination

Protein concentration was determined in accordance with Bradford [25] using BSA (bovine serum albumin) as a standard. SDS-PAGE was carried out on slabs (10 × 5.5 cm) of 12% polyacrylamide gel, in line with Laemmli's method [26]. The samples were denatured for 10 min at 95°C in the presence of 10% SDS and 0.5% 2-mercaptoethanol.

### Enzyme characterization

The thermodependency of the enzyme activity was determined by incubating 5 μl of the purified BgaL enzyme (0.12 mg ml^-1^) in a 0.02 M sodium phosphate buffer, pH 7.3, with 100 μl of ONPG (3 mM), for 2 min at temperatures ranging from 0 to 70°C. Reactions halted by the addition of 100 μl Na_2_CO_3 _(1 M) and then hydrolysis of the *o*-nitrophenyl group was then detected at 405 nm. The thermostability of the BgaL enzyme was determined by incubating it at 20, 30, 40, and 50°C, removing aliquots for up to 120 min. The enzyme activity was assayed at 20°C in the same manner as used for the thermodependency of enzyme activity assays.

One unit (U) of the activity denoted 1 μmol of *o*-nitrophenol liberated from the substrate (ONPG) in 1 min and under the standard reaction conditions. (20 mM sodium phosphate buffer pH 7.3 and 20°C).

The optimum pH was determined by assaying activity of the BgaL enzyme at a 10 mM Britton-Robinson buffer, with pH values ranging from 3.0 to 11.0. Enzyme activity was measured at 20°C and as described above. The pH stability profiles for the enzyme activity of the BgaL was determined by an initial incubation of the enzyme for 2 h, at 20°C and in 10 mM Britton-Robinson buffer solutions (pH 4.0-9.0), followed by determination of the activity under conditions described above.

Requirements for metal ions (10 mM) and selected reagents: 10 mM EDTA, 10 mM DTT, 10 mM glutathione (oxidized form), 10 mM 2-mercaptoethanol and 10 mM urea solutions, were determined under standard conditions.

The substrate specificity of BgaL enzyme was estimated using 12 chromogenic substrates: *o*-nitrophenyl-β-D-galactopyranoside (ONPG), *p*-nitrophenyl-β-D-galactopyranoside (PNPG), *p*-nitrophenyl-β-D-galacturonide, *p*-nitrophenyl-β-L-arabinopyranoside, *p*-nitrophenyl-β-D-cellobioside, *p*-nitrophenyl-β-D-mannopyranoside, *p*-nitrophenyl-β-D-glucopyranoside, *p*-nitrophenyl-α-D-galactopyranoside, *p*-nitrophenyl-β-D-fucopyranoside, *p*-nitrophenyl-β-D-xylopyranoside, and *p*-nitrophenyl-β-D-glucuronide each at a concentration of 5 mM. Activity was measured at 20°C and as described above.

The kinetic parameters of the freshly purified enzyme were determined at 10, 20 and 30°C, and the reaction rate with ONPG (1.0-5.0 mM) and lactose (1.0-5.0 mM) as substrates was determined, respectively. The lactose concentration after enzymatic reaction was determined using Liquick Cor-Glucose kit (Cormay) to measure the concentration of glucose released during lactose hydrolysis.

The efficiency of 1U of *Paracoccus *sp. 32d β-D-galactosidase, in the hydrolysis of the lactose in milk (10 ml) at 10°C was monitored by means of HPLC analysis using Aminex HPX-87H column (Bio-Rad), 5 mM H_2_SO_4 _as a mobile phase and Agilent 1200 Series Refractive Index Detector. 1% solutions of glucose, galactose and lactose were used as standards.

All experiments (above-mentioned) were done in triplicate.

### Nucleotide sequences accession numbers

The 16S rDNA and β-D-galactosidase gene sequences reported in this work have been deposited in the GenBank database and assigned the Accession Nos. GU111730.1 and GU111731.1, respectively.

## List of abbreviations used

aa: amino-acid residues; Cys: cysteine; GSSG: oxidized glutathione; GH2: glycoside hydrolase family 2; IBT: Institute of Technical Biochemistry; HPLC: high-performance liquid chromatography; IPTG: isopropyl β-D-1-thiogalactopyranoside; ONPG: *o*-nitrophenyl-β-D-galactopyranoside; PNPG: *p*-nitrophenyl-β-D-galactopyranoside; X-Gal: 5-bromo-4-chloro-3-indolyl-β-D-galactopyranoside.

## Competing interests

The authors declare that they have no competing interests.

## Authors' contributions

AWW performed the cloning experiments, protein expression and purification experiments, characterized the BgaL enzyme and was involved in drafting the manuscript, HC analyzed the data, drafted the manuscript and coordinated the study, MW assisted with the protein purification and participated in drafting the manuscript, KKT carried out the HPLC experiments, PH isolated *Paracoccus *sp. strain 32d, JK conceived and assisted in coordinating its realization. All the authors have read and have approved the final manuscript.
